# Resource Barrier Versus Resource Drain: Adult Attachment Differentially Moderates the Impact of Social Support on Well-Being Through Need Satisfaction

**DOI:** 10.3390/bs16071170

**Published:** 2026-07-11

**Authors:** Xi Chen, Azlina Mohd Khir, Hanina Halimatusaadiah Hamsan, Nik Ahmad Sufian Burhan

**Affiliations:** Faculty of Human Ecology, Universiti Putra Malaysia, Serdang 43400, Selangor, Malaysia

**Keywords:** perceived social support, subjective well-being, basic psychological need satisfaction, adult attachment, moderated mediation

## Abstract

Perceived social support consistently predicts subjective well-being (SWB), yet the underlying psychological mechanisms and boundary conditions remain insufficiently understood. Integrating Conservation of Resources (COR) theory with Self-Determination Theory (SDT), this study tested a moderated mediation model in which basic psychological need satisfaction (autonomy, competence, relatedness) mediates the social support–SWB relationship, with adult attachment orientations (anxiety, avoidance) as moderators. A sample of 488 Chinese university students (52.0% female; M_age_ = 20.15) completed self-report measures. Parallel mediation analysis showed that all three needs significantly mediated the social support–SWB link, with competence as the strongest mediator. Moderated mediation analyses showed that attachment avoidance significantly weakened the indirect effect through competence satisfaction, whereas attachment anxiety, despite significant negative main effects, did not moderate any pathway. These findings suggest that avoidance may function as a “resource barrier” that attenuates the internalization of support into need satisfaction, whereas anxiety may operate as a “resource drain,” associated with depleted baseline resources while the support-uptake mechanism remains intact. This study advances theory by proposing distinct functional roles for attachment dimensions within an integrated COR-SDT framework. Practical implications for attachment-informed, need-supportive interventions among university students are discussed.

## 1. Introduction

As a cornerstone of positive psychology, Subjective Well-being (SWB) is conceptualized as a multifaceted construct that encompasses how individuals appraise their lives through both cognitive judgments and emotional experiences (Diener, 1984; Diener et al., 2018). University students, who typically fall within the developmental period of emerging adulthood (ages 18–25; Arnett, 2000), face distinctive challenges including academic pressures, career uncertainties, and identity exploration that render them particularly vulnerable to decrements in well-being (E. Sheldon et al., 2021; Wang & Tong, 2025; Zheng & Yan, 2024). This developmental period is characterized by heightened instability and identity formation, during which peer relationships become increasingly salient and the satisfaction of basic psychological needs for autonomy, competence, and relatedness takes on particular developmental significance (Arnett, 2000; Luyckx et al., 2009). Understanding the determinants and underlying processes of well-being during this critical period is therefore important for both theoretical advancement and evidence-based intervention.

Despite robust evidence that perceived social support predicts well-being (S. Cohen & Wills, 1985; Huang & Zhang, 2022; Yeo et al., 2025), two critical questions remain insufficiently addressed. The first concerns mechanism: through what psychological processes social support may be linked to enhanced well-being. While mediators such as self-esteem and coping strategies have been explored (W. Liu et al., 2016; Ozer, 2024), these investigations often lack a coherent theoretical framework that specifies why external social resources promote internal psychological flourishing. According to the nutriment hypothesis proposed within Self-Determination Theory (Ryan & Deci, 2000, 2017), the fulfillment of fundamental psychological needs is the primary driver of well-being. Despite this theoretical foundation, few studies have explicitly modeled all three needs as simultaneous mediators to explain the association between social support and well-being. The second question concerns boundary conditions: for whom is social support most effective? Individuals vary considerably in the degree to which they benefit from available support (Lakey & Orehek, 2011; Yeo et al., 2025), yet the factors underlying this heterogeneity remain underexplored. Attachment theory (Mikulincer & Shaver, 2016) provides a compelling basis for this variation. Specifically, anxiety and avoidance reflect qualitatively different interpersonal strategies that may condition the support–well-being link through distinct pathways rather than functioning as interchangeable risk factors.

The present study addresses these questions within an integrated framework combining COR theory (Hobfoll et al., 2018) and SDT (Ryan & Deci, 2017). Specifically, the present study focuses on how perceived social support contributes to basic psychological need satisfaction and, in turn, subjective well-being, as well as the conditions under which this process is strengthened or weakened by individual attachment orientations. Accordingly, we propose and test a moderated mediation model in which: (1) need satisfaction (autonomy, competence, relatedness) serves as the mechanism linking perceived support to well-being; (2) adult attachment orientations moderate the conversion of social support into need satisfaction; and (3) the buffering effect of attachment is specific to the resource internalization process (indirect path) or generalizes to the direct outcome (direct path). This study makes three contributions. First, it investigates need satisfaction as a theoretically grounded mediator connecting social support with well-being, moving beyond atheoretical mediator selection toward a principled account rooted in SDT’s nutriment hypothesis. Second, it examines whether attachment avoidance and anxiety play functionally distinct roles in conditioning the social support–well-being relationship, a distinction we characterize as “resource barrier” versus “resource drain.” Third, it illustrates the potential value of COR-SDT integration by operationalizing COR’s abstract resource concepts through SDT’s well-validated psychological constructs.

### 1.1. Perceived Social Support and Subjective Well-Being

Perceived social support, defined as individuals’ appraisals of assistance accessible through their social connections (Cobb, 1976; Dambi et al., 2018; Zimet et al., 1988), consistently predicts SWB. Meta-analytic evidence confirms medium to large effect sizes for this association across diverse populations and cultural contexts (Yeo et al., 2025). This relationship has been attributed to both stress-buffering mechanisms that help individuals cope with life stressors (S. Cohen & Wills, 1985; Alvarado-García et al., 2026) and main-effect pathways that fulfill fundamental needs for belonging regardless of stress levels (Holt-Lunstad, 2022).

Among Chinese university students in particular, such support positively predicts life satisfaction, positive affect, and overall psychological functioning (Huang & Zhang, 2022; Zhou & Xie, 2025), with longitudinal evidence indicating that this association persists over time (Sharifian & Grühn, 2018). Moreover, the interpersonal dynamics underlying the social support–well-being association may be shaped by culturally specific norms governing help-seeking and reciprocity, particularly in Chinese collectivist contexts (X. Li & Bian, 2024). Demographic factors may also be relevant to the social support–well-being relationship. Research suggests that women tend to perceive and mobilize more social support than men (Taylor et al., 2000), and gender differences in subjective well-being have been documented, with young men often reporting higher well-being than young women (Diener et al., 2018). Additionally, in the Chinese context, household registration (hukou) status has been associated with disparities in access to social resources and well-being between rural and urban populations (Knight & Gunatilaka, 2010). Accordingly, gender and household registration were included as covariates in all analyses to account for these potential confounding effects. Drawing on these theoretical and empirical insights, we hypothesize:

**H1.**  *Perceived social support positively predicts subjective well-being.*

### 1.2. The Mediating Role of Basic Psychological Need Satisfaction

According to SDT (Deci & Ryan, 2000; Ryan & Deci, 2017), environmental factors promote well-being primarily by fulfilling three fundamental psychological needs: autonomy, competence, and relatedness. Although all three needs independently contribute to well-being (Vansteenkiste et al., 2020), their relative associations with well-being may differ across developmental contexts. Among university students, competence satisfaction may be particularly strongly associated with well-being, as academic achievement and professional development represent central concerns during emerging adulthood (Arnett, 2000; Ryan & Deci, 2020). Relatedness satisfaction is also expected to be closely linked to well-being given the heightened salience of peer relationships during this period (Baumeister & Leary, 1995), while autonomy satisfaction fosters well-being through a sense of volitional functioning (Ryan & Deci, 2017). Importantly, need satisfaction represents the proximal psychological mechanism through which external resources become functionally incorporated into the self (Ryan & Deci, 2000, 2017; Vansteenkiste et al., 2020).

Perceived social support is theoretically linked to all three need dimensions (X. Chen et al., 2025). It may foster relatedness by providing tangible evidence of connection and belonging (Baumeister & Leary, 1995; Maas et al., 2022), enhance competence through instrumental assistance and positive feedback that affirm one’s effectiveness (Bandura, 1997; Vansteenkiste et al., 2020), and support autonomy by expanding resources and options for volitional goal pursuit (Deci & Ryan, 2000; Ryan & Deci, 2017). Although existing studies have shown that need satisfaction mediates the relationship between environmental supports and well-being (Patrick et al., 2007; B. Chen et al., 2015), few have simultaneously tested all three need dimensions as parallel mediators of the social support–SWB relationship. This gap is significant, as examining these differential pathways could clarify how social support promotes well-being. Based on SDT’s theoretical predictions and supporting empirical evidence, we propose:

**H2.**  *Basic psychological need satisfaction (autonomy, relatedness, competence) mediates the relationship between perceived social support and subjective well-being.*

### 1.3. The Moderating Role of Adult Attachment

Adult attachment is characterized by two dimensions: anxiety (concerns about rejection and abandonment) and avoidance (discomfort with intimacy and reliance on others). These dimensions reflect stable cognitive–affective schemas rooted in early caregiving experiences (Bowlby, 1982; Hazan & Shaver, 1987) that shape how individuals perceive, seek, and utilize interpersonal resources (Brennan et al., 1998; Mikulincer & Shaver, 2023). Both dimensions are associated with lower well-being across cultures (MacDonald & Park, 2022; Zhang et al., 2022). Crucially for the present study, the two dimensions are associated with qualitatively different strategies for processing social support, suggesting that they may condition the support–well-being relationship through distinct mechanisms.

Individuals with elevated attachment avoidance employ deactivating strategies marked by discomfort with closeness and compulsive self-reliance (Mikulincer & Shaver, 2023). They actively resist accepting support to maintain independence, which may substantially hinder social support from satisfying psychological needs. This resistance may be particularly salient for competence satisfaction, as avoidant individuals tend to rely on defensive self-enhancement rather than external validation (Richardson & Boag, 2024; Richardson et al., 2023), making it unlikely that they will translate interpersonal feedback into feelings of effectiveness. By contrast, individuals high in attachment anxiety employ hyperactivating strategies marked by intense proximity-seeking and hypervigilance to rejection signals (Mikulincer & Shaver, 2023). Unlike their avoidant counterparts, anxiously attached individuals do not block social support input; they remain highly receptive to interpersonal resources due to their chronic need for reassurance (Messina et al., 2023; Snyder et al., 2024). Their difficulties may therefore stem from chronic resource depletion, as persistent worry and rumination consume psychological resources, rather than from an inability to process available support.

These differential mechanisms imply that the two attachment dimensions may moderate the social support-need satisfaction link in distinct ways. While attachment theory posits that secure attachment facilitates effective support utilization whereas insecure attachment may hinder this process (Mikulincer & Shaver, 2009), empirical studies further suggest that the two insecurity dimensions may differentially shape how individuals benefit from available support (Costa-Cordella et al., 2022; Dark-Freudeman et al., 2020; Lenzo et al., 2022). However, these studies have not proposed a theoretical account of why the two dimensions moderate this association differently, nor have the specific need-satisfaction pathways through which this differential moderation may operate been examined. Based on these considerations, we propose:

**H3.**  *Attachment anxiety and avoidance moderate the paths from perceived social support to basic psychological need satisfaction.*

### 1.4. Theoretical Integration

The present research synthesizes COR theory and SDT in developing a framework that addresses the explanatory limitations of each perspective individually. Specifically, COR provides the interactional architecture, while SDT provides the functional mechanism.

To elucidate this integration, we first turn to the resource dynamics outlined by COR theory. COR theory posits that resources aggregate in “resource caravans,” such that greater initial resource endowment enhances capacity for further gains, whereas resource scarcity increases vulnerability to continued loss (Hobfoll, 1989, 2011). This dynamic implies that existing internal resources should condition the effectiveness of external resource utilization, a proposition termed the “resource synergy” corollary (Hobfoll, 2011; Halbesleben et al., 2014). However, COR theory does not specify the psychological mechanism through which this synergy operates (Halbesleben et al., 2014), nor does it distinguish whether qualitatively different internal deficits constrain resource utilization through different pathways. SDT addresses the first gap by identifying need satisfaction as the proximal mechanism linking environmental supports to well-being (Ryan & Deci, 2017). However, SDT’s predictions regarding need satisfaction primarily concern main effects and do not formally theorize about multiplicative interactions between environmental supports and individual orientations (Ryan & Deci, 2017).

**Figure 1:**
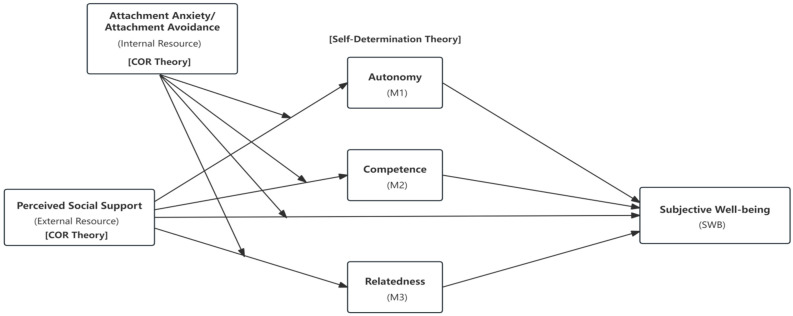
The Conceptual Model of the Study.

The integration produces three predictions unavailable to either theory alone. First, COR’s resource synergy effect should operate specifically through need satisfaction, yielding a moderated mediation model rather than simple moderation. Second, combining COR’s interaction logic with SDT’s tripartite need structure predicts that qualitatively different internal deficits (i.e., avoidant deactivation versus anxious hyperactivation) should constrain different need-satisfaction pathways. This reasoning suggests pathway-specific moderation patterns rather than uniform attenuation across all needs. Third, moderation should be localized at the indirect path linking social support-to-need satisfaction rather than at the direct path to well-being, because need satisfaction represents the specific process through which resource internalization occurs. These predictions jointly inform the moderated mediation model tested in this study (Figure 1).

## 2. Materials and Methods

### 2.1. Participants and Procedure

A total of 500 Chinese university students were recruited via multi-stage random sampling across Anhui Province, mainland China. Sampling proceeded through four stages: universities, colleges within universities, classes within colleges, and individual students selected by ID numbers. After excluding invalid questionnaires (incomplete data or patterned responses), we retained 488 valid responses (response rate: 97.6%). Participants’ ages ranged from 18 to 23 years (M = 20.15, SD = 1.42). Additional demographic information (gender, hukou, grade) appears in Table 1. This study was approved by the Ethics Committee for Research Involving Human Subjects of Universiti Putra Malaysia (Ref: JKEUPM-2025-245). Trained researchers administered questionnaires in randomly selected classes. After explaining the study’s purpose and ensuring anonymity, students provided written consent. Completion required approximately 20 min. Compensation was 10 RMB per participant.

### 2.2. Measures

#### 2.2.1. Perceived Social Support

The Multidimensional Scale of Perceived Social Support (MSPSS; Zimet et al., 1988; Chinese version: Yan & Zheng, 2006) measured perceived social support. The 12-item instrument assesses support from three sources: family (e.g., ‘My family really tries to help me’), friends (e.g., ‘I can count on my friends when things go wrong’), and significant others (e.g., ‘There is a special person in my life who cares about my feelings’). Participants responded on 7-point scales (1 = strongly disagree, 7 = strongly agree). Higher scores indicate greater perceived social support. The MSPSS has demonstrated excellent psychometric properties in Chinese populations (Yan & Zheng, 2006). Current study reliability was high: Cronbach’s α = 0.92.

#### 2.2.2. Adult Attachment

The Experiences in Close Relationships Scale-Chinese Version (ECR-C; T. Li & Kato, 2006) assessed adult attachment orientations. The 36-item instrument comprises two 18-item subscales measuring attachment anxiety (e.g., ‘I worry about being abandoned’) and attachment avoidance (e.g., ‘I prefer not to show a partner how I feel deep down’). Participants responded on 7-point scales (1 = strongly disagree, 7 = strongly agree). Subscale scores were computed as the mean of the 18 items in each dimension. Higher scores indicate greater attachment insecurity. The ECR-C has shown strong reliability and validity in Chinese samples (T. Li & Kato, 2006). Current study reliabilities were excellent: Cronbach’s α = 0.91 for attachment anxiety and Cronbach’s α = 0.88 for attachment avoidance.

#### 2.2.3. Basic Psychological Need Satisfaction

The Basic Psychological Need Satisfaction Scale (BPNSS; Deci & Ryan, 2000; Chinese version: J. Liu et al., 2013) measured satisfaction of three basic psychological needs. The 21-item instrument comprises three subscales assessing autonomy (7 items; e.g., ‘I feel like I am free to decide for myself how to live my life’), competence (6 items; e.g., ‘Most days I feel a sense of accomplishment from what I do’), and relatedness (8 items; e.g., ‘People in my life care about me’). Participants responded on 7-point scales (1 = not at all true, 7 = very true). Higher scores indicate greater need satisfaction. The Chinese version has demonstrated good psychometric properties (J. Liu et al., 2013). Current study reliabilities were acceptable: Cronbach’s α = 0.78 for autonomy, Cronbach’s α = 0.82 for competence, and Cronbach’s α = 0.85 for relatedness.

#### 2.2.4. Subjective Well-Being

Subjective well-being (SWB) was operationalized as a composite of cognitive and affective components assessed by two measures (Diener, 1984). The Satisfaction with Life Scale (SWLS; Diener et al., 1985; Chinese version: Xiong & Xu, 2009) assessed global life satisfaction (5 items; e.g., ‘In most ways my life is close to my ideal’) on a 7-point scale (1 = strongly disagree, 7 = strongly agree). The Positive and Negative Affect Schedule (PANAS; Watson et al., 1988; Chinese version: Huang et al., 2003) measured emotional experiences (20 items) on a 5-point scale, comprising positive affect (PA; e.g., ‘enthusiastic,’ ‘interested’) and negative affect (NA; e.g., ‘distressed,’ ‘upset’). Following established practice (Diener, 1984; K. M. Sheldon & Elliot, 1999), a global composite score was computed as SWB = Z_SWLS_ + Z_PA_ − Z_NA_. This operationalization treats SWB as a unified construct in which cognitive and affective components contribute jointly to an overall assessment of well-being. Higher composite scores indicate greater overall subjective well-being. Current study reliabilities were satisfactory: Cronbach’s α = 0.84 for life satisfaction, Cronbach’s α = 0.87 for positive affect, and Cronbach’s α = 0.86 for negative affect.

### 2.3. Data Analysis

Statistical analyses were conducted in multiple phases using SPSS 26.0, AMOS 29.0, and Hayes’ (2022) PROCESS macro. Initially, descriptive statistics, internal consistency coefficients (Cronbach’s α), and bivariate correlations among all study variables were examined. A confirmatory factor analysis (CFA) was then conducted in AMOS 29.0.

For hypothesis testing, the PROCESS macro was selected over full latent-variable structural equation modeling. Although SEM can estimate the entire model simultaneously and explicitly account for measurement error, the primary objective of the present study was to test theoretically specified conditional indirect effects and their boundary conditions. The proposed model involves moderated parallel mediation with three simultaneous mediators, a configuration for which PROCESS Model 8 is specifically designed, providing bias-corrected bootstrap confidence intervals for conditional indirect effects and indices of moderated mediation (Hayes, 2022). To mitigate the limitation of using observed variables, the measurement model was validated via CFA, and satisfactory composite reliability (CR) and convergent validity (AVE) were confirmed, supporting the construct validity of the composite measures used in subsequent analyses.

To test the mediating role of basic psychological needs (Hypothesis 2), we employed PROCESS Model 4 to estimate the indirect effects linking social support to subjective well-being via autonomy, competence, or relatedness simultaneously. The moderated mediation models (Hypothesis 3) were tested using PROCESS Model 8, which examines moderation of both the mediation paths and the direct effect. Given that the two attachment dimensions are theorized to exert qualitatively different moderating effects, two separate Model 8 analyses were conducted: one with avoidance as the moderator and another with anxiety, with the alternate dimension entered as a covariate in each analysis. This approach enables independent evaluation of each dimension’s moderation pattern and the computation of separate indices of moderated mediation, essential for distinguishing the hypothesized ‘resource barrier’ and ‘resource drain’ mechanisms.

Predictor variables underwent z-score transformation prior to analysis for ease of coefficient interpretation (Aiken & West, 1991). Bias-adjusted bootstrap estimation (5000 replications) computed 95% CIs surrounding all indirect pathways and conditional indirect pathways. Significance was inferred when 95% CIs omitted zero. Simple slope analyses examined significant interactions at one standard deviation below versus above the mean of the moderator. Demographic controls (gender, household registration) were applied in all analyses.

## 3. Results

### 3.1. Measurement Model and Common Method Bias Test

We employed CFA in AMOS 29.0 to verify the factorial distinctiveness of the nine measured constructs and to establish composite reliability (CR) and average variance extracted (AVE) in support of subsequent observed-variable analyses. The CFA also provided a preliminary assessment of common method bias (Podsakoff et al., 2003). The hypothesized nine-factor structure (comprising Attachment Anxiety, Attachment Avoidance, Perceived Social Support, Autonomy, Competence, Relatedness, Life Satisfaction, Positive Affect, and Negative Affect) exhibited satisfactory fit (χ^2^/df = 3.69, CFI = 0.927, TLI = 0.911, RMSEA = 0.074, 90% CI [0.070, 0.078]). Standardized loadings were statistically significant (*p* < 0.001), ranging from 0.615 to 0.954. Additionally, Composite Reliability (CR) spanned 0.808 to 0.942, while Average Variance Extracted (AVE) spanned 0.588 to 0.844, confirming excellent convergent validity.

To address common method bias concerns inherent in self-reported data, we contrasted the nine-factor structure against a single-factor alternative wherein all items loaded onto one latent construct (Podsakoff et al., 2003). The single-factor solution yielded markedly poor fit (χ^2^/df = 21.30, CFI = 0.378, TLI = 0.327, RMSEA = 0.204, 90% CI [0.200, 0.208]), indicating that a single method factor does not account for the covariance structure in the data. This single-factor comparison serves only as a preliminary diagnostic and does not definitively rule out shared-method variance.

### 3.2. Descriptive Statistics and Correlations

Descriptive statistics and bivariate correlations are presented in Table 2. As anticipated, perceived social support was positively correlated with all three basic psychological needs (autonomy, competence, relatedness) and subjective well-being (*p* < 0.01). Conversely, both attachment anxiety and avoidance showed negative correlations with social support, need satisfaction, and subjective well-being.

### 3.3. Testing Moderated Mediation Models

To test the proposed conceptual model, the PROCESS macro for SPSS (Hayes, 2022) was employed. The analysis proceeded in two steps: first, Model 4 was used to examine the parallel mediation effects of basic psychological needs (Social Support predicting Need Satisfaction predicting Subjective Well-being); second, Model 8 was used to examine whether attachment orientations (anxiety and avoidance) moderated both the first stage of the mediation and the direct path. Gender and household registration (hukou) were included as covariates in all analyses. All continuous predictors were standardized prior to analysis, yielding standardized regression coefficients (β) that serve as effect size indicators comparable across predictors.

#### 3.3.1. Testing the Mediation Effect

Before examining the moderated mediation models, the mediating role of basic psychological needs (Hypothesis 2) was tested using PROCESS Model 4. Gender and household registration were included as covariates. The results are summarized in Table 3.

Results indicated that social support significantly predicted all three mediators: autonomy (β = 0.28, *p* < 0.001), competence (β = 0.21, *p* < 0.001), and relatedness (β = 0.28, *p* < 0.001). These needs, in turn, predicted subjective well-being: autonomy (β = 0.43, *p* < 0.001), competence (β = 0.73, *p* < 0.001), and relatedness (β = 0.44, *p* < 0.001).

Bias-corrected bootstrap analysis (5000 resamples) showed a significant total indirect effect of social support on subjective well-being through the three needs (Effect = 0.39, 95% CI [0.318, 0.475]). Crucially, all three specific indirect pathways were significant, as their 95% confidence intervals did not include zero: Autonomy (Effect = 0.12, 95% CI [0.067, 0.175]), Competence (Effect = 0.15, 95% CI [0.096, 0.223]), and Relatedness (Effect = 0.12, 95% CI [0.081, 0.166]). The direct effect of social support on subjective well-being remained significant after controlling for the mediators (β = 0.12, *p* = 0.004), indicating a partial mediation. Because all variables were standardized prior to analysis, the specific indirect effects serve as standardized effect size indicators directly comparable across pathways. Competence satisfaction emerged as the strongest mediator (Effect = 0.15), followed by relatedness (Effect = 0.12) and autonomy (Effect = 0.12). These findings fully support Hypothesis 2.

#### 3.3.2. The Moderating Role of Attachment Avoidance

**Figure 2:**
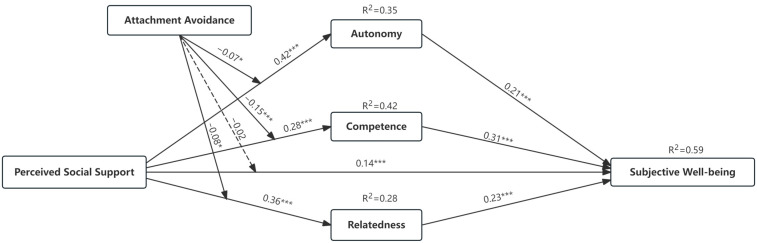
Statistical path diagram of the moderated mediation model with Attachment Avoidance as the moderator. Note. Values are standardized coefficients (β). * *p* < 0.05, *** *p* < 0.001. Solid lines represent significant paths (*p* < 0.05); dashed lines represent non-significant paths. R^2^ values indicate the variance explained for each endogenous variable.

To examine the specific moderating role of attachment avoidance, we conducted a moderated mediation analysis using PROCESS Model 8. In this model, perceived social support was entered as the independent variable, subjective well-being as the dependent variable, and attachment avoidance as the moderator. Crucially, all three basic psychological needs (autonomy, competence, and relatedness) were entered simultaneously as parallel mediators to control for their intercorrelations. Additionally, attachment anxiety, along with gender and household registration, was included as a covariate to isolate the unique effects of avoidance. The detailed results are presented in Table 4, and the full statistical path diagram appears in Figure 2. As shown in the figure, solid lines on the interaction paths indicate significant moderating effects.

**Figure 3:**
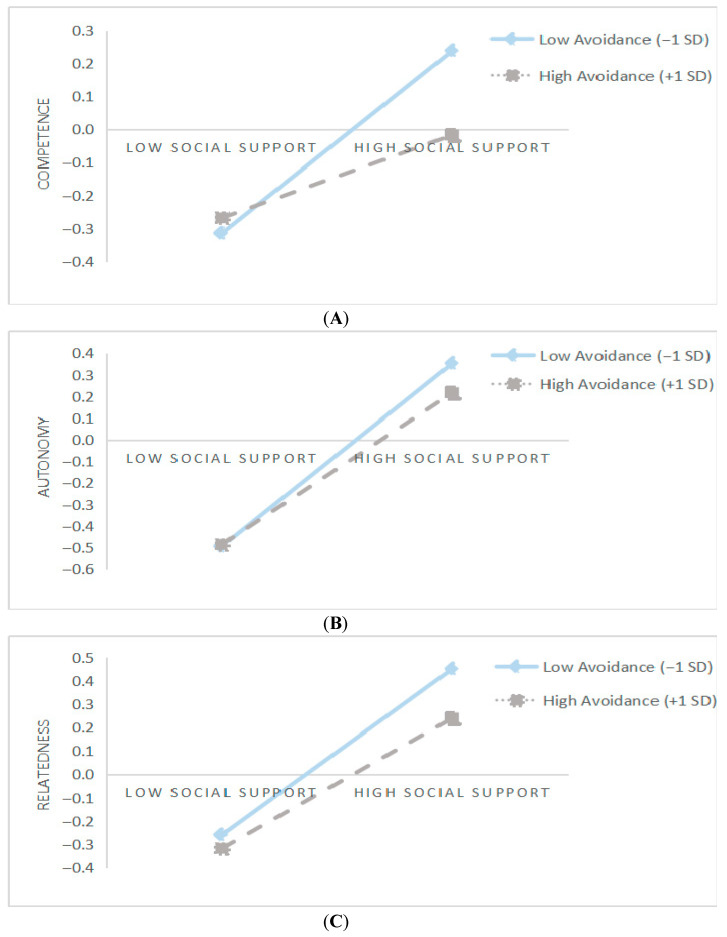
Simple slopes of perceived social support predicting basic psychological need satisfaction at low (−1 SD) and high (+1 SD) levels of attachment avoidance. (**A**): competence satisfaction; (**B**): autonomy satisfaction; (**C**): relatedness satisfaction.

The results showed a distinct pattern of moderation across the three need-satisfaction pathways. First, regarding the competence pathway, the interaction between social support and attachment avoidance was highly significant (β = −0.15, *p* < 0.001, ΔR^2^ = 0.03). Simple slope analyses (see Figure 3A) indicated that the positive effect of social support on competence satisfaction was robust among individuals with low attachment avoidance (−1 SD; Effect = 0.43, *p* < 0.001). However, this effect was substantially weaker for those with high attachment avoidance (+1 SD; Effect = 0.13, *p* = 0.011). The index of moderated mediation for the competence pathway was significant (Index = −0.046, 95% CI [−0.073, −0.022]), indicating that attachment avoidance was associated with a significantly weaker indirect effect of social support on well-being through competence satisfaction.

Second, for autonomy and relatedness, the interaction terms were also significant (Autonomy: β = −0.07, *p* = 0.019, ΔR^2^ = 0.01; Relatedness: β = −0.08, *p* = 0.018, ΔR^2^ = 0.01), indicating that higher attachment avoidance was associated with a weaker positive association between social support and the satisfaction of these needs. However, the indices of moderated mediation for these two pathways included zero (Autonomy: 95% CI [−0.036, 0.002]; Relatedness: 95% CI [−0.042, 0.001]). This suggests that while the first-stage moderation was statistically present, the conditional indirect effects through autonomy and relatedness were not as robustly moderated by avoidance as the competence pathway was after controlling for attachment anxiety.

Finally, regarding the direct path, the interaction between social support and attachment avoidance on subjective well-being was not significant (β = −0.02, *p* = 0.538). This pattern suggests that the link between attachment avoidance and the attenuation of social support’s association with well-being is concentrated in the need-satisfaction pathways, particularly competence, rather than in the direct pathway.

As shown in Table 4, the models explained substantial variance in each outcome, with R^2^ values ranging from 0.28 (relatedness) to 0.59 (subjective well-being), all representing large effects by J. Cohen’s (1988) conventions.

To further interpret the significant interaction effects of attachment avoidance on the three basic psychological needs reported in Table 4, we conducted simple slope analyses at low (−1 SD) and high (+1 SD) levels of attachment avoidance. The full conditional effects are presented in Table 5.

First, regarding competence satisfaction (the pathway most strongly moderated by avoidance), the interaction pattern is depicted in Figure 3A. Results showed that for students with low attachment avoidance, perceived social support was a strong and positive predictor of competence satisfaction (Effect = 0.43, *p* < 0.001). However, for those with high attachment avoidance, this positive relationship was substantially attenuated (Effect = 0.13, *p* = 0.011).

Second, for autonomy satisfaction, the simple slope analysis (Figure 3B) showed a similar buffering pattern. The positive association between social support and autonomy was more pronounced for individuals with low attachment avoidance (Effect = 0.50, *p* < 0.001) compared to those with high attachment avoidance (Effect = 0.35, *p* < 0.001). This suggests that while social support remains positively associated with autonomy satisfaction for individuals high in avoidance, the strength of this association is significantly weaker than for their less avoidant peers.

Finally, regarding relatedness satisfaction, as illustrated in Figure 3C, attachment avoidance was also associated with a weaker beneficial association between social support and relatedness. Specifically, the positive association between social support and relatedness was stronger for low-avoidance individuals (Effect = 0.43, *p* < 0.001) than for high-avoidance individuals (Effect = 0.28, *p* < 0.001).

#### 3.3.3. The Moderating Role of Attachment Anxiety

Following the same analytical strategy, we examined the moderating role of attachment anxiety using PROCESS Model 8. In this analysis, attachment anxiety served as the moderator, while attachment avoidance was included as a covariate to control for its effects.

Contrary to the findings for attachment avoidance, the results (presented in Table 6) indicated that attachment anxiety did not significantly moderate the relationship between perceived social support and any of the three basic psychological needs: Autonomy (β = −0.01, *p* = 0.834, ΔR^2^ = 0.00), Competence (β = 0.04, *p* = 0.217, ΔR^2^ = 0.00), and Relatedness (β = −0.01, *p* = 0.717, ΔR^2^ = 0.00). Similarly, the interaction effect on the direct path from social support to subjective well-being was non-significant (β = −0.02, *p* = 0.417). Consequently, the indices of moderated mediation for all three pathways included zero, indicating the absence of a significant conditional indirect effect (Autonomy: 95% CI [−0.020, 0.017]; Competence: 95% CI [−0.016, 0.036]; Relatedness: 95% CI [−0.024, 0.022]).

It is important to note, however, that attachment anxiety showed significant negative main effects on all three needs and subjective well-being (*p*s < 0.001). This suggests that while attachment anxiety may be associated with generally depleted psychological resources (consistent with a “resource drain” pattern), it does not appear to function as a “resource barrier” that attenuates the effectiveness of social support when it is available.

## 4. Discussion

This research investigated an integrative model connecting perceived social support with subjective well-being (SWB) in Chinese university students, positioning basic psychological need satisfaction as the mediating process and adult attachment orientations as contextual moderators. Synthesizing COR and SDT frameworks, we tested a moderated mediation framework examining how, when, and under what conditions social support may be associated with well-being. Three principal results emerged: (1) satisfaction of basic psychological needs (autonomy, competence, and relatedness) partially mediated the relationship between social support and SWB; (2) attachment avoidance significantly moderated support-to-need-satisfaction pathways, particularly for competence; and (3) attachment anxiety, though negatively related to need satisfaction and well-being, did not moderate support effectiveness. We discuss these findings regarding their theoretical contributions, practical applications, and future research directions.

### 4.1. The Mediating Role of Basic Psychological Need Satisfaction

All three basic psychological needs (autonomy, competence, and relatedness) significantly mediated the link from perceived social support to SWB. This result is consistent with SDT’s nutriment hypothesis (Ryan & Deci, 2017; Vansteenkiste et al., 2020), which posits that environmental factors promote well-being primarily through fulfilling basic psychological needs. It also extends B. Chen et al.’s (2015) finding that need satisfaction mediates environment-well-being links across cultural contexts, by specifically testing this mechanism within the social support domain.

Among the three needs, competence satisfaction emerged as the strongest mediator, followed by relatedness and autonomy. The prominence of competence satisfaction is noteworthy and may reflect the developmental context of university students, for whom academic and professional competence is a central concern (R. Cohen & Katz, 2024; Ryan & Deci, 2020). Social support may enhance competence satisfaction by providing instrumental assistance, informational guidance, and positive feedback that affirm students’ capabilities and effectiveness. This interpretation aligns with Bandura’s (1997) social cognitive framework, which emphasizes how social persuasion and vicarious experience, both components of social support, contribute to self-efficacy.

That relatedness satisfaction also played a significant mediating role is consistent with the theoretical expectation that social support most directly addresses the need for belonging and connection (Baumeister & Leary, 1995; Martela et al., 2023). Perceived social support signals that one is valued and cared for by others, which may directly address the need for meaningful interpersonal bonds. Similarly, the indirect effect through autonomy satisfaction was significant, supporting the SDT proposition that supportive social environments can enhance volitional functioning by providing resources that expand personal choice (Ryan & Deci, 2017).

The significant direct association between social support and SWB after controlling for need satisfaction indicates partial mediation, suggesting that additional mechanisms may operate alongside need satisfaction. These may include emotion regulation processes, self-esteem enhancement, or stress-buffering effects that do not necessarily operate through need satisfaction pathways (S. Cohen & Wills, 1985; Holt-Lunstad, 2022). Investigating these complementary mechanisms in future studies would yield a more complete understanding of the social support–well-being relationship.

Importantly, the parallel mediation design employed in this study represents a methodological advance over prior investigations that tested need dimensions separately. By estimating all three indirect effects simultaneously, our approach controls for the shared variance among the three needs and provides more precise estimates of each specific pathway (Hayes, 2022). This approach showed that all three needs make unique contributions, underscoring the importance of treating them as distinct yet complementary mechanisms rather than collapsing them into a single composite.

Across models, female students reported significantly lower subjective well-being than male students (Table 4 and Table 6), consistent with research indicating gender differences in well-being during emerging adulthood (Diener et al., 2018). Female students also reported lower relatedness satisfaction (Table 6), suggesting that gender may differentially shape the fulfillment of specific psychological needs. While gender was included as a covariate rather than a focal variable in the present study, these patterns warrant attention in future research, which could employ multi-group analyses to examine whether the proposed mediating and moderating mechanisms operate differently across gender.

### 4.2. The Differential Moderating Roles of Attachment Avoidance and Attachment Anxiety

#### 4.2.1. Attachment Avoidance as a “Resource Barrier”

Attachment avoidance significantly moderated the indirect effect of social support on well-being through competence satisfaction, as indicated by a significant index of moderated mediation. This finding supports a key theoretical prediction derived from COR theory’s resource synergy hypothesis (Hobfoll, 2011) and attachment theory’s account of deactivating strategies (Mikulincer & Shaver, 2023). According to attachment theory, individuals high in attachment avoidance tend to resist accepting interpersonal support and maintain self-reliance, deactivating their attachment system and distancing themselves from relational resources (Mikulincer & Shaver, 2023). This deactivating orientation appears to function as what we term a “resource barrier”, an internal psychological condition that may impede the effective utilization of external resources for psychological need satisfaction.

The finding that the competence pathway was the most strongly moderated by avoidance is theoretically meaningful. Competence satisfaction is theorized to involve the internalization of external feedback and support as evidence of one’s own effectiveness (Deci & Ryan, 2000; Vansteenkiste et al., 2020). However, for avoidant individuals, this internalization process appears to be disrupted. This pattern is consistent with the “defensive self-esteem” account of avoidant attachment (Cassidy et al., 2009; Richardson & Boag, 2024). Avoidantly attached individuals often maintain an inflated yet fragile self-image that relies on compulsive self-reliance. For them, accepting social support may imply weakness or dependency, which poses a psychological threat to their defensive self-view. As a result, they tend to dismiss the relevance of others’ feedback and insist on self-generated evidence of worth (Richardson et al., 2023; Mikulincer & Shaver, 2023). This defensive appraisal may function as a “resource barrier,” attenuating the process through which social support would otherwise enhance feelings of effectiveness.

This resource barrier may be further intensified by culturally specific interpersonal dynamics. In Chinese society, social interactions are governed by norms of ‘Mianzi’ (Face) and ‘Renqing’ (Reciprocity), whereby accepting support from others often creates implicit social debts that must be repaid (Hwang, 2012; X. Li & Bian, 2024). For avoidant individuals who already experience discomfort with dependency, the prospect of incurring such reciprocal obligations may further deter them from accepting interpersonal resources, reinforcing the deactivating tendency. This culturally compounded barrier suggests that moderation patterns observed here may reflect both universal attachment dynamics and context-specific relational costs that amplify avoidance-related resistance to support.

For the autonomy and relatedness pathways, the first-stage interaction terms were statistically significant, indicating that attachment avoidance attenuated the association between social support and these two needs. However, the indices of moderated mediation for both pathways included zero, meaning that the conditional indirect effects through autonomy and relatedness were not significantly moderated after controlling for attachment anxiety. This pattern suggests that while avoidance may dampen the initial resource conversion process across need dimensions, the full moderated mediation chain from social support through need satisfaction to well-being was only robustly established for the competence pathway. The consistent direction of negative interaction effects across all three need dimensions is suggestive of a broader attenuating tendency, but this interpretation should be regarded as a theoretical extrapolation.

Notably, attachment avoidance did not moderate the support–SWB direct association, suggesting that avoidance may impede well-being primarily by attenuating the psychological internalization process (need satisfaction) rather than by weakening direct stress-buffering or belonging-related mechanisms. This specificity of moderation at the indirect pathway level aligns with SDT’s proposition that need satisfaction serves as a critical proximal mechanism linking environmental factors to well-being (Ryan & Deci, 2017; Vansteenkiste et al., 2020), a pattern consistent with the theoretical integration proposed in this study.

#### 4.2.2. Attachment Anxiety as a “Resource Drain”

Attachment anxiety did not significantly moderate the relationship between perceived social support and any of the three basic psychological needs, or the direct path to subjective well-being. Although this null interaction finding is inconsistent with the unified moderation predicted in Hypothesis 3, it is congruent with the theoretical rationale outlined earlier. Hyperactivating strategies do not entail resistance to support input and may therefore yield a different moderation pattern relative to avoidance.

Unlike the “resource barrier” pattern observed for avoidance, we interpret the pattern of results for attachment anxiety as consistent with what may be termed a “resource drain” effect. This interpretation is rooted in the fundamental difference between deactivating and hyperactivating strategies (Mikulincer & Shaver, 2016, 2023). Anxiously attached individuals employ hyperactivating strategies, characterized by an intense craving for proximity and reassurance. Consequently, they are unlikely to block or resist the intake of social support; rather, they tend to actively seek it for reassurance and validation (Snyder et al., 2024; Mikulincer & Shaver, 2023). This interpretation is consistent with the finding that the positive association between social support and need satisfaction remained significant regardless of anxiety level, with none of the interaction terms reaching statistical significance. At the same time, their chronic worry, rumination, and hypervigilance may act as a constant drain on psychological resources, potentially contributing to a lower baseline of well-being even when support is available. Under this account, anxiety may deplete the baseline reservoir of psychological resources while leaving the support-uptake mechanism relatively unimpaired. This distinction extends COR theory’s resource synergy hypothesis (Hobfoll et al., 2018) by suggesting that different forms of internal resource deficit may constrain external resource utilization through different pathways. It should be noted, however, that the “resource drain” characterization represents a theoretical inference derived from the observed combination of significant negative main effects and null moderation effects, rather than a mechanism directly tested in the present design.

This differential pattern aligns with accumulating evidence indicating that the two attachment dimensions relate to psychological outcomes through distinguishable pathways. Zhang et al.’s (2022) comprehensive meta-analytic review found that both attachment dimensions were associated with poorer mental health, but that anxiety showed larger effect sizes than avoidance on both positive and negative outcomes, indicating differences in the strength and nature of their influence. At the process level, Messina et al. (2023) showed that these differential associations reflect distinct emotion regulation profiles, with attachment anxiety linked to an exaggerated dependence on others for emotion regulation (hyperactivation) and attachment avoidance linked to a pseudo-autonomous reliance on intrapersonal strategies (deactivation). The present findings extend this line of research by suggesting that these differential mechanisms may operate not only in predicting well-being outcomes but also in conditioning the effectiveness of external resources.

From a COR theory perspective, the distinction between resource barriers and resource drains offers a potential refinement of Hobfoll et al.’s (2018) resource synergy hypothesis by suggesting that not all internal resource deficits may constrain external resource utilization in the same way. Some internal deficits (such as avoidance) may actively attenuate resource conversion, while others (such as anxiety) may deplete existing resources without necessarily interfering with the benefits of new resource inputs.

**Figure 4:**
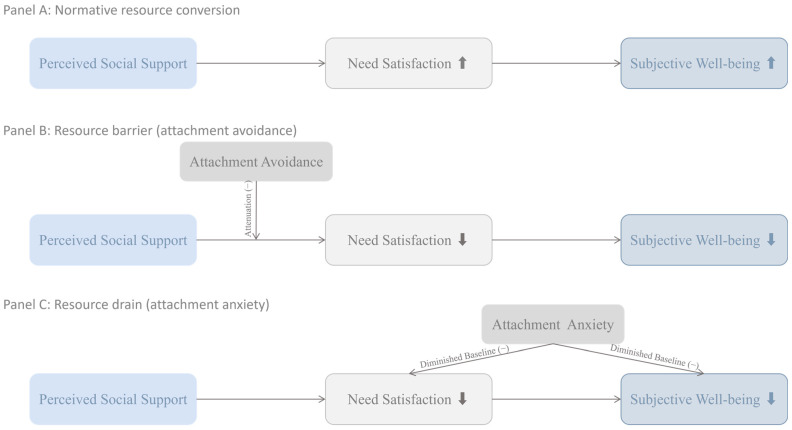
Theoretical illustration of the “Resource Barrier” versus “Resource Drain” mechanisms. Horizontal arrows represent directional pathways between variables; vertical arrows within boxes indicate the direction of change (↑ = increase; ↓ = decrease).

Figure 4 provides a theoretical illustration contrasting these two mechanisms. Panel A depicts the normative resource conversion process, Panel B illustrates how attachment avoidance attenuates the translation of social support into need satisfaction (Resource Barrier), and Panel C illustrates how attachment anxiety lowers need satisfaction levels through its main effects without moderating the conversion pathway (Resource Drain).

### 4.3. Theoretical Contributions

The present study makes three primary theoretical advances. First, it highlights need satisfaction as a specific, theoretically grounded mediating mechanism linking social support to well-being. Although prior studies have examined various mediators of this relationship, such as self-esteem, emotion regulation, as well as coping strategies (Thoits, 2011; Razurel et al., 2013; Vowels et al., 2023), few have simultaneously tested multiple need dimensions within a unified theoretical framework that specifies why external resources should promote internal flourishing. By embedding the mediation within SDT’s nutriment hypothesis (Ryan & Deci, 2017; Vansteenkiste et al., 2020), the present study offers a principled and integrative account linking perceived social support to well-being through the satisfaction of basic psychological needs, which SDT identifies as critical nutrients for psychological flourishing.

Second, the study offers preliminary evidence suggesting that attachment avoidance and anxiety may operate through different mechanisms in conditioning the social support–well-being relationship. The clearest empirical support is for the competence pathway, where attachment avoidance significantly moderated the conditional indirect effect. The ‘resource barrier’ versus ‘resource drain’ distinction represents a theoretically grounded framework for understanding heterogeneity in social support effectiveness. This framework goes beyond the common practice of treating attachment dimensions as parallel predictors and instead proposes distinct functional roles for each dimension within the resource utilization process, though its generalizability across all need-satisfaction pathways requires further investigation.

Third, the findings provide initial support for the COR-SDT integration proposed, yielding a pattern of results consistent with its three unique predictions: resource synergy operating through need satisfaction (moderated mediation), qualitatively different deficits constraining different pathways (avoidance selectively moderating competence), and moderation localized at the indirect path. This integration addresses a recognized limitation of COR theory (Halbesleben et al., 2014; Hobfoll et al., 2018), namely its insufficient specification of psychological pathways linking resources to outcomes. It also suggests that the combined framework may generate testable predictions beyond what either theory offers independently.

### 4.4. Practical Implications

The present findings suggest several practical implications for mental health interventions targeting university students. The mediation by autonomy, competence, and relatedness suggests that support programs could benefit from simultaneously cultivating all three needs. Rather than merely increasing social support, the focus could shift toward delivering support in need-supportive ways at the institutional level. For example, universities could adopt autonomy-supportive teaching practices that offer meaningful choices and acknowledge students’ perspectives (Reeve & Cheon, 2021), implement structured peer mentoring programs that provide both social integration and a sense of belonging (Gehreke et al., 2024), and create inclusive campus communities through collaborative learning environments (Ryan & Deci, 2020; Vansteenkiste et al., 2020). These universal, group-based approaches address all three needs without requiring identification of individual psychological profiles, thereby minimizing the risk of stigmatization.

Second, the finding that attachment avoidance is associated with reduced translation of social support into need satisfaction has important implications for counseling practice. Students with avoidant attachment patterns may not benefit from conventional support-enhancement interventions given their reliance on deactivating strategies that prioritize compulsive self-reliance over interdependence. For these students, interventions that initially focus on building awareness of avoidant tendencies and gradually cultivating comfort with interpersonal dependence may be more effective than simply increasing support availability (Mikulincer & Shaver, 2023). Emotion-focused approaches that help avoidant individuals recognize and process their relational needs may be particularly valuable in this regard (Johnson, 2019). At the group level, psychoeducational workshops on attachment awareness could also help students recognize how their relational patterns influence their capacity to benefit from available support.

Third, the finding that the positive association between social support and need satisfaction remains intact for anxiously attached students, despite their lower overall well-being, provides an optimistic message for intervention. Increasing perceived social support through structured peer mentoring programs, strengthening friend networks, and providing consistent, reliable access to counseling services are strategies that may improve well-being across the student population, and may be particularly beneficial for students who experience elevated psychological distress but remain receptive to interpersonal resources.

### 4.5. Limitations and Future Directions

This study has notable constraints. Most critically, its cross-sectional structure limits causal inferences (Maxwell & Cole, 2007). Although the theoretical model specifies directional relationships, the data cannot rule out reverse causation or reciprocal effects. Prospective work should employ longitudinal and experience-sampling methods to establish temporal ordering and capture dynamic processes.

Second, all major variables were assessed through concurrent self-report measures in a single session, raising the possibility that shared-method variance may partially inflate the observed associations. Although the single-factor CFA comparison confirmed that the constructs are empirically distinguishable, factorial separability does not preclude common source effects (Podsakoff et al., 2003). Future investigations should incorporate diverse measurement strategies, such as peer ratings of social support or behavioral indicators of need satisfaction, to provide convergent validation beyond self-report assessment.

Third, the sample consisted exclusively of Chinese university students in Anhui Province, which may limit generalizability. Although SDT (Ryan & Deci, 2017) and attachment theory (Mikulincer & Shaver, 2016) are proposed as culturally universal frameworks, the specific patterns of moderation may vary across cultural contexts with different norms regarding support-seeking and reliance on social networks (Kim et al., 2008). Cross-cultural replication with samples from Western and other non-Western populations would strengthen the generalizability of the findings.

Fourth, the PROCESS macro operates on observed composite scores and does not partition measurement error from true construct variance as latent-variable structural equation modeling does. This reliance on observed variables may attenuate interaction effects, potentially yielding conservative estimates of the moderation patterns reported here (Marsh et al., 2004). Future studies could employ latent moderated structural equation approaches (Maslowsky et al., 2015) with larger samples to obtain more precise estimates of these conditional indirect effects.

Fifth, the present study operationalized subjective well-being as a global composite of standardized life satisfaction, positive affect, and negative affect scores. Although this approach is consistent with established practice (Diener, 1984) and provides a parsimonious assessment of overall well-being, it does not permit examination of whether the predictors relate differently to the cognitive versus affective components of well-being. Future research could model these components separately to determine whether the mediating and moderating patterns identified here operate uniformly across well-being dimensions or vary by component.

Beyond addressing these limitations, several directions merit investigation. Differentiating among support sources (family, friends, significant others; Zimet et al., 1988) and support types (emotional, instrumental, informational; S. Cohen & Wills, 1985) could reveal whether attachment avoidance more strongly moderates certain forms of support than others. Additionally, person-centered analyses (e.g., latent profile analysis) could identify distinct attachment configurations (e.g., secure, preoccupied, dismissive, fearful; Bartholomew & Horowitz, 1991), thereby complementing the present variable-centered findings and illuminating whether specific attachment subtypes show unique patterns of support utilization.

## 5. Conclusions

This research suggests that perceived social support is positively associated with subjective well-being in Chinese university populations through a pattern consistent with the satisfaction of autonomy, competence, and relatedness needs. Furthermore, the results indicate that adult attachment orientations may function distinctly within this process: avoidance may operate as a “resource barrier,” associated with reduced effectiveness of social support in promoting need satisfaction, particularly for competence, while anxiety may operate as a “resource drain,” associated with diminished baseline well-being without attenuating the benefits of available support. By synthesizing COR and SDT, the present study proposed a framework for understanding the potential interplay between internal dispositions and external resources associated with subjective well-being, with implications for designing culturally sensitive, need-supportive interventions for university students.

## Figures and Tables

**Table 1:** Demographic Characteristics of the Participants (N = 488).

Variable	Category	Frequency	Percentage
Gender	Male	234	48.0
	Female	254	52.0
Household Registration (Hukou)	Urban	367	75.2
	Rural	121	24.8
Grade Level	Freshman (Grade 1)	122	25.0
	Sophomore (Grade 2)	120	24.6
	Junior (Grade 3)	122	25.0
	Senior (Grade 4)	124	25.4

**Table 2:** Means, Standard Deviations, and Correlations among Study Variables.

Variable	M	SD	1	2	3	4	5	6	7
1. Attachment Anxiety	3.58	1.04	1						
2. Attachment Avoidance	3.45	0.89	0.11 *	1					
3. Social Support	56.63	11.25	−0.12 **	−0.47 **	1				
4. Autonomy	28.67	6.49	−0.35 **	−0.30 **	0.50 **	1			
5. Competence	26.68	6.26	−0.52 **	−0.29 **	0.39 **	0.62 **	1		
6. Relatedness	40.95	7.08	−0.30 **	−0.32 **	0.44 **	0.35 **	0.39 **	1	
7. Subjective Well-being	0.00	2.24	−0.47 **	−0.27 **	0.43 **	0.59 **	0.64 **	0.50 **	1

Note. N = 488. * *p* < 0.05, ** *p* < 0.01. SWB was computed as Z_SWLS_ + Z_PA_ − Z_NA_, where Z denotes within-sample standardization. Because each standardized component has a mean of zero, the resulting composite also has a mean of zero by construction. All other variables are reported as raw scores.

**Table 3:** Parallel Mediation Analysis of Basic Psychological Needs in the Relationship Between Social Support and Subjective Well-being.

**Effects**	**β**	**SE**	**t**	***p***	**95% LLCI**	**95% ULCI**
Total Effect (Support → SWB)	0.51	0.04	11.64	<0.001	0.426	0.599
Direct Effect (Support → SWB)	0.12	0.04	2.93	0.004	0.039	0.198
**Indirect Effects**	**Effect**	**Boot SE**			**Boot LLCI**	**Boot ULCI**
Total Indirect Effect	0.39	0.04			0.318	0.475
Ind 1: via Autonomy	0.12	0.03			0.067	0.175
Ind 2: via Competence	0.15	0.03			0.096	0.223
Ind 3: via Relatedness	0.12	0.02			0.081	0.166

Note. N = 488. Covariates: Gender and Hukou. Bootstrap sample size = 5000. All coefficients are standardized. CI = Confidence Interval.

**Table 4:** Moderated Mediation Analysis of Perceived Social Support on Subjective Well-being via Need Satisfaction (Moderator: Attachment Avoidance).

Predictors	Model 1: Autonomy		Model 2: Competence		Model 3: Relatedness		Model 4: Subjective Well-Being	
	β	SE	β	SE	β	SE	β	SE
**Controls**								
Gender	0.12	0.08	0.10	0.07	−0.15	0.08	−0.12 *	0.06
Hukou	−0.08	0.09	−0.10	0.08	0.06	0.09	−0.11	0.07
**Covariate**								
Attachment Anxiety	−0.28 ***	0.04	−0.44 ***	0.04	−0.21 ***	0.04	−0.13 ***	0.04
**Main Effects**								
Social Support (X)	0.42 ***	0.04	0.28 ***	0.04	0.36 ***	0.04	0.14 ***	0.04
Attachment Avoidance (W)	−0.06	0.04	−0.11 **	0.04	−0.13 **	0.04	0.04	0.03
**Mediators**								
Autonomy							0.21 ***	0.04
Competence							0.31 ***	0.04
Relatedness							0.23 ***	0.03
**Interaction**								
Support × Avoidance	−0.07 *	0.03	−0.15 ***	0.03	−0.08 *	0.03	−0.02	0.03
ΔR^2^	0.01		0.03		0.01		0.00	
R^2^	0.35		0.42		0.28		0.59	
*F*	42.64 ***		57.60 ***		30.76 ***		75.05 ***	
**Index of Moderated Mediation**	**Index**	**95% CI**	**Index**	**95% CI**	**Index**	**95% CI**		
Autonomy	−0.015	[−0.036, 0.002]						
Competence			−0.046	[−0.073, −0.022]				
Relatedness					−0.018	[−0.042, 0.001]		

Note. N = 488. Values are standardized coefficients (β). * *p* < 0.05, ** *p* < 0.01, *** *p* < 0.001. Gender: 1 = Male, 2 = Female; Hukou: 1 = Urban, 2 = Rural. Covariate “Attachment Anxiety” was included in all models to isolate the effect of avoidance. ΔR^2^ represents the incremental variance explained by the interaction term beyond the main effects.

**Table 5:** Conditional Effects of Perceived Social Support on Basic Psychological Need Satisfaction at Low (−1 SD) and High (+1 SD) Levels of Attachment Avoidance.

Need Dimension	Avoidance Level	Effect	SE	t	*p*	95% LLCI	95% ULCI
Autonomy	Low (−1 SD)	0.50	0.05	9.44	<0.001	0.393	0.599
Autonomy	High (+1 SD)	0.35	0.05	6.78	<0.001	0.250	0.454
Competence	Low (−1 SD)	0.43	0.05	8.62	<0.001	0.330	0.525
Competence	High (+1 SD)	0.13	0.05	2.55	0.011	0.029	0.221
Relatedness	Low (−1 SD)	0.43	0.06	7.82	<0.001	0.323	0.540
Relatedness	High (+1 SD)	0.28	0.05	5.12	<0.001	0.172	0.387

Note. N = 488. Effects represent the conditional effect of perceived social support on each need dimension at low and high levels of attachment avoidance, controlling for attachment anxiety, gender, and household registration.

**Table 6:** Moderated Mediation Analysis of Perceived Social Support on Subjective Well-being via Need Satisfaction (Moderator: Attachment Anxiety).

Predictors	Model 1: Autonomy		Model 2: Competence		Model 3: Relatedness		Model 4: Subjective Well-Being	
	β	SE	β	SE	β	SE	β	SE
**Controls**								
Gender	0.11	0.08	0.07	0.07	−0.16 *	0.08	−0.12 *	0.06
Hukou	−0.09	0.09	−0.11	0.08	0.05	0.09	−0.11	0.07
**Covariate**								
Attachment Avoidance	−0.06	0.04	−0.10 *	0.04	−0.13 **	0.04	0.04	0.03
**Main Effects**								
Social Support (X)	0.42 ***	0.04	0.28 ***	0.04	0.35 ***	0.05	0.14 ***	0.04
Attachment Anxiety (W)	−0.30 ***	0.04	−0.47 ***	0.04	−0.23 ***	0.04	−0.13 ***	0.04
**Mediators**								
Autonomy							0.21 ***	0.04
Competence							0.31 ***	0.04
Relatedness							0.24 ***	0.03
**Interaction**								
Support × Anxiety	−0.01	0.03	0.04	0.03	−0.01	0.03	−0.02	0.02
ΔR^2^	0.00		0.00		0.00		0.00	
R^2^	0.34		0.39		0.27		0.59	
*F*	41.24 ***		50.56 ***		29.51 ***		75.12 ***	
**Index of Moderated Mediation**	**Index**	**95% CI**	**Index**	**95% CI**	**Index**	**95% CI**		
Autonomy	−0.001	[−0.020, 0.017]						
Competence			0.012	[−0.016, 0.036]				
Relatedness					−0.003	[−0.024, 0.022]		

Note. N = 488. Values are standardized coefficients (β). * *p* < 0.05, ** *p* < 0.01, *** *p* < 0.001. Gender: 1 = Male, 2 = Female; Hukou: 1 = Urban, 2 = Rural. Covariate “Attachment Avoidance” was included in all models to isolate the unique effect of anxiety. ΔR^2^ represents the incremental variance explained by the interaction term beyond the main effects.

## Data Availability

The data presented in this study are available on request from the corresponding author due to privacy and ethical restrictions.

## Abbreviations

The following abbreviations are used in this manuscript:

SWB	Subjective Well-Being
COR	Conservation of Resources
SDT	Self-Determination Theory
MSPSS	Multidimensional Scale of Perceived Social Support
ECR-C	Experiences in Close Relationships Scale-Chinese Version
BPNSS	Basic Psychological Need Satisfaction Scale
SWLS	Satisfaction with Life Scale
PANAS	Positive and Negative Affect Schedule
PA	Positive Affect
NA	Negative Affect
CFA	Confirmatory Factor Analysis
CR	Composite Reliability
AVE	Average Variance Extracted
SEM	Structural Equation Modeling
CI	Confidence Interval
LLCI	Lower Limit Confidence Interval
ULCI	Upper Limit Confidence Interval
